# Histone H3 dopaminylation in ventral tegmental area underlies heroin-induced transcriptional and behavioral plasticity in male rats

**DOI:** 10.1038/s41386-022-01279-4

**Published:** 2022-01-29

**Authors:** Sasha L. Fulton, Swarup Mitra, Ashley E. Lepack, Jennifer A. Martin, Andrew F. Stewart, Jacob Converse, Mason Hochstetler, David M. Dietz, Ian Maze

**Affiliations:** 1 Nash Family Department of Neuroscience, Friedman Brain Institute, Icahn School of Medicine at Mount Sinai, New York, NY 10029 USA; 2 Department of Pharmacology and Toxicology, Program in Neuroscience, University at Buffalo, Buffalo, NY 14214 USA; 3 Department of Pharmacological Sciences, Icahn School of Medicine at Mount Sinai, New York, NY 10029 USA; 4 Howard Hughes Medical Institute, Icahn School of Medicine at Mount Sinai, New York, NY 10029 USA

**Keywords:** Epigenetics and behaviour, Addiction

## Abstract

Persistent transcriptional events in ventral tegmental area (VTA) and other reward relevant brain regions contribute to enduring behavioral adaptations that characterize substance use disorder. Recent data from our laboratory indicate that aberrant accumulation of the newly discovered histone post-translational modification (PTM), H3 dopaminylation at glutamine 5 (H3Q5dop), contributes significantly to cocaine-seeking behavior following prolonged periods of abstinence. It remained unclear, however, whether this modification is important for relapse vulnerability in the context of other drugs of abuse, such as opioids. Here, we showed that H3Q5dop plays a critical role in heroin-mediated transcriptional plasticity in midbrain regions, particularly the VTA. In rats undergoing abstinence from heroin self-administration (SA), we found acute and persistent accumulation of H3Q5dop in VTA. Attenuation of H3Q5dop during abstinence induced persistent changes in gene expression programs associated with neuronal signaling and dopaminergic function in heroin abstinence and led to reduced heroin-seeking behavior. Interestingly, the observed changes in molecular pathways after heroin SA showed significant yet reversed overlap with the same genes altered in cocaine SA. These findings establish an essential role for H3Q5dop, and its downstream transcriptional consequences, in heroin-induced functional plasticity in VTA.

## Introduction

Opioid Use Disorder (OUD) has dramatically increased to epidemic proportions in recent years, with heroin dependence growing more than 50% over the last decade and accounting for more drug-related deaths [1] than any other substance of abuse [2–5]. Clinically, OUD is characterized by compulsive drug-seeking behaviors that can persist for years and extend over the course of a lifetime, with one of the highest risks of relapse among substance use disorders [1–5]. Relapse vulnerability is a key diagnostic feature of addiction, and represents a highly complex process involving multiple circuits mediated by dynamic alterations in cellular plasticity within the mesolimbic reward system, where aberrant dopamine neurotransmission promotes maladaptive reward-processing and persistent deficits in motivated behaviors. Given the recurring, long-lasting nature of OUD, the field has begun to place great emphasis on drug-induced epigenetic regulation as a potential key mechanism in opioid addiction and relapse [6–10]. To date, however, our understanding of the causal mechanisms that mediate drug-induced transcriptional and cellular plasticity remains incomplete, particularly in the relatively understudied ventral tegmental area (VTA), which contains critical dopaminergic projections to reward-processing striatal regions.

Recently, our laboratory demonstrated that in addition to modulating downstream synaptic activity as a neurotransmitter, dopamine itself plays a direct role in epigenetically coordinating gene expression within these dopaminergic cells in the VTA [11]. We previously found that certain monoamine neurotransmitters, including dopamine, can serve as donors for covalent modifications on specific residues of substrate proteins (termed monoaminylations) through an enzymatic process mediated by the writer-protein Transglutaminase 2 (TGM2) [12]. Among the identified substrate proteins for monoaminylation is histone H3, which is modified at glutamine 5 (Q5) on its N-terminal tail. At this H3Q5 site, specific histone monoaminylation marks (e.g., serotonylation or dopaminylation) were found to alter gene expression, either directly or through combinatorial interactions with other nearby histone PTMs, suggesting that these modifications may constitute an important epigenetic regulatory mechanism in monoaminergic neuromodulation [12, 13]. In a recent report, we confirmed that histone H3 dopaminylation (H3Q5dop) has a key functional role in cocaine-mediated transcriptional plasticity, dopaminergic neurotransmission, and cocaine-seeking behavior in a rodent model of drug relapse vulnerability [11]. Given the importance of H3Q5dop in mediating cocaine-induced transcriptional and behavioral plasticity, here we investigated whether functional regulation by H3Q5dop represents an important mechanism in dependence for other addictive substances such as heroin, or if this phenomenon was specific to cocaine.

All drugs of abuse are thought to elicit changes in dopaminergic neurotransmission from VTA, although different classes of substances act on this system through distinct cellular mechanisms. In addition, the magnitude and temporal dynamics of dopamine transmission vary widely between drugs, even within class, highlighting the importance of delineating substance-specific mechanisms in order to develop targeted therapeutic strategies to address the molecular changes associated with each drug [14]. In the present study, we investigated specific roles for H3Q5dop in heroin-induced VTA transcriptional plasticity and drug-seeking behavior in a rodent model of relapse vulnerability. We found that abstinence from heroin SA resulted in the accumulation of H3Q5dop in VTA, though this increase occurs more rapidly in response to heroin vs. cocaine abstinence. We further showed that aberrant H3Q5dop dynamics in VTA contribute significantly to heroin-induced gene expression and drug-seeking behaviors. These findings indicate that H3Q5dop may represent a conserved epigenetic substrate underlying addiction to distinct classes of abused drugs (e.g., cocaine and heroin), though the regulation of gene expression programs that lead to altered DA output from VTA may be specific to heroin’s mechanism of action.

## Materials and methods

### Animals

Male Sprague-Dawley rats (Envigo; 250–300 g) were singly housed during self-administration experimental procedures, and maintained on a 12 h reverse light/dark cycle with food and water available *ad libitum*. All procedures were done in accordance with NIH guidelines and the Institutional Animal Care and Use Committees of the Icahn School of Medicine at Mount Sinai and the State University of New York at Buffalo.

### Jugular catheterization

For both heroin SA and saline SA, rats were implanted with chronic indwelling jugular catheters, as previously reported [15, 16]. Rats were given 5 d of recovery, during which time the catheters were flushed with 0.2 mL of a solution containing enrofloxacin (4 mg/mL) and heparin saline (50 IU/mL in 0.9% sterile saline) to preserve catheter patency. All animals received an i.v. infusion of ketamine hydrochloride (0.5 mg/kg in 0.05 mL) one day prior to behavioral training to verify catheter patency. Rats that responded with a loss of muscle tone and righting reflexes were considered patent and were included in cohorts for behavioral training.

### Heroin Self-Administration and Viral surgeries

Rats were allowed to recover from jugular catheterization surgery for 5 d, and then were trained to SA heroin (0.02 mg/kg/inf) or saline (as a non-reinforcement control) in operant chambers for 3 h each day for a period of 10 d. A response in the active hole nose poke resulted in an intravenous injection of heroin (0.02 mg/kg/inf) or saline using a fixed ratio 1 (FR1) schedule of reinforcement on day 1 that was increased to FR3 on day 3 and maintained for the remainder of the experiment [15]. Responses in the inactive hole resulted in no programmed consequences. Following training sessions, catheters were flushed, and rats were returned to their colony rooms. For viral manipulations, all rats self-administered heroin or saline, and subsequently received intra-VTA bilateral injections of either AAV2-GFP (empty vector control), AAV2-H3.3WT, or AAV2-H3.3Q5A on AD11 (see below for additional information on surgery coordinates).

### Cue-induced seeking test

For the cue-induced seeking behavior, on AD30, rats were returned to operant chambers for a testing duration of 1 h. During the test duration, rats were subjected to extinction-like conditions whereby responses in an active hole delivered only the cue light previously associated with drug availability. The total number of active responses in the active hole was an operational measure of heroin-seeking behavior [17, 18].

### Western blotting and antibodies

VTA tissues were collected from heroin SA rats (2 mm punches) on AD1, AD14 or AD30 and frozen. Nuclear fractions were purified via homogenization in Buffer A containing 10 mM HEPES (pH 7.9), 10 mM KCl, 1.5 mM MgCl_2_, 0.34 M sucrose, 10% glycerol, 1 mM EDTA, 1X protease inhibitor cocktail and 1X phosphostop inhibitor. After homogenization, 0.1% TritonX-100 was added, samples were incubated on ice for 30 min and then centrifuged for 5 min at 1300 × *g* at 4 °C. Supernatants (i.e., cytosolic fractions) were discarded, and nuclear pellets were resuspended in Buffer A to remove any remaining cytosolic contamination, followed by centrifugation for 5 min at 1300 × *g* at 4 °C. Supernatants were then discarded and pellets resuspended and sonicated in sample buffer containing 0.3 M sucrose, 5 mM HEPES, 1% SDS and 1X protease inhibitor cocktail. Protein concentrations were measured with the DC protein assay kit (BioRad), and 10–15 ug of protein was loaded onto 4–12% NuPage BisTris gels (Invitrogen) for electrophoresis. Proteins were next transferred to nitrocellulose membranes and blocked for 1 h in 5% milk in PBS + 0.1% Tween 20 (PBST), followed by primary antibody incubation for two days at 4 °C. The following antibodies were used in this manuscript: rabbit anti-H3Q5dop (1:200, ABE2588; Millipore), rabbit anti-H3K4me3Q5dop (1:500, ABE2590; Millipore), rabbit anti-H3K4me3 (1:1000, lot #: GR273043-6, Abcam ab8580), mouse anti-Tgm2 (1:500, lot #: Gr3188112-4, Abcam ab2386) and rabbit anti-H3 (1:50000, lot #: GR293151-1, Abcam ab1791). Membranes were then washed 3X in PBST (10 min) and incubated for 1 h with horseradish peroxidase conjugated anti-rabbit (BioRad 170-6515, lot #: 64033820) or anti-mouse (GE Healthcare UK Limited NA931V, lot #: 9814763) secondary antibodies (1:10000; 1:50000 for anti-H3 antibody, BioRad) in 5% milk/PBST at RT. After three more washes with PBST, bands were detected using enhanced chemiluminescence (ECL; Millipore). Densitometry was used to quantify protein bands using Image J Software (NIH), and proteins were normalized to total H3. H3 measurements were obtained from the same membrane as the protein of interest after stripping the primary antibody for 10 min in Restore Stripping Buffer (Thermo #21059).

### AAV constructs and viral infusion

Adeno-associated virus H3.3 constructs [empty vs. wildtype (WT) vs. (Q5A)-Flag-HA] were generated and validated, as previously described [11, 12, 19]. All three vectors contain a GFP fluorescent tag to allow visualization of the injection site during tissue dissection. The H3.3Q5A vector used in these studies, which does not affect adjacent H3K4me3, inhibits the expression of all modifications on Q5. However, appreciable signal for other monoaminyl marks (e.g., H3Q5ser) is not observed in VTA vs. H3Q5dop [11]. After 24 h following the last day of heroin vs. saline SA (day 11), animals were anaesthetized with a ketamine/xylazine solution (60/5 mg/kg) i.p., positioned in a stereotaxic frame (Kopf instruments) and 1 μl of viral construct was infused bilaterally into VTA using the following coordinates; anterior-posterior (AP) −4.9, medial-lateral [20] +2.1, dorsal-ventral [21] −7.6. After surgery, rats received meloxicam (1 mg/kg) s.c. and topical antibiotic treatments for ~3 days. Of note, previous reports comparing heroin cue-seeking behavior at AD14 vs. AD30 have found no significant differences in heroin-seeking behavior between these two extended abstinence time points [22]. Furthermore, the present experiments demonstrated robust heroin-seeking behavior at AD30 (see Fig. 3). Therefore, to ensure that we captured gene expression changes at a timepoint corresponding to maximal transgene expression from our AAV serotype, (which begins at 7 d post-infusion and increases to maximal expression at 28d) all tissue collections or behavioral testing commenced 30 d after viral surgery to allow for maximal expression of the viral constructs, as previously validated [11].

### RNA isolation and sequencing

Rat brains were removed and immediately snap frozen. Brains were then sectioned to 100 uM thick coronal slices on a cryostat at −20 °C, and 2 mm punches were dissected from virally infected VTA using GFP-fluorescence illuminated via a NIGHTSEA Dual Fluorescent Protein flashlight (#DFP-1). Total RNA was then extracted from punches using Trizol (Thermo Fisher #15596026)-chloroform, followed by cleanup and elution into RNAase-free water using Qiagen minelute (#74204) columns, as per manufacturer instructions. Following elution, RNA samples were enriched for mRNA via polyA tail selection beads, and mRNA libraries were prepared using the Illumina Truseq RNA Library Prep Kit V2 (#RS-122-2001). Libraries were pooled and sequenced on the Illumina Novaseq platform. RNA-seq data were pre-processed and analyzed, as previously described [11]. Briefly, FastQC (Version 0.72) was performed on the concatenated replicate raw sequencing reads from each library to ensure minimal PCR duplication and sequencing quality. Reads were aligned to the rn6 genome using HISAT2 (Version 2.1.0) and annotated against Ensembl v90. Multiple-aligned reads were removed, and remaining transcript reads were counted using featurecounts (Version 2.0.1). Technical variation between samples and batch effects were calculated and normalized using the RUVr model of RUVseq (Version 4.1.1). DESEQ2 [23] (Version 2.11.40.6) was then used to normalize read counts between the groups, and to perform pairwise differential expression analyses.

Differentially expressed (DEx) genes were defined at FDR cutoff <0.05, Log2FC ± 0.2. Threshold free Rank-Rank Hypergeometric Overlap (RRHO) maps were generated to visualize transcriptome-wide gene expression concordance patterns as previously described [24], using RRHO2 (Version 1.0). DE genes were overlapped (Venny) to identify the subset of DE genes reversed between heroin H3.3Q5A and both heroin empty and heroin H3.3 WT. Odds Ratio analyses were carried out on DE gene lists using the *GeneOverlap* R package version 1.26.0 [25]. Processed read count data for cocaine comparisons was extracted from our previous publication [11], deposited in the National Center for Biotechnology Information Gene Expression Omnibus database under accession number GSE124055.

In order to determine if differential gene expression comparisons showed significant overlaps with annotated gene sets, we performed GO Enrichment Analyses using the EnrichR package (Version 3.0) against EnrichR’s gene set databases Panther Pathway, GO Biological Process 2021, and GO Molecular Function 2021. Enrichments were ranked by adj. *p* value [26], computed using Fisher’s exact test followed by Benjamini-Hochberg (BH) correction. Multivariant plots display top ranked gene sets, Y-axis = rank from 1–5 for each term, plotted according to -log10(adj.*p* value) on the X-axis, and the combined score of the enrichment (bubble size). The combined score is derived from both the *p* value and the z-score (deviation from expected rank based on gene set size) calculated by the following formula: c = ln(p) * z. Fill color represents the database source of terms. For Fig. S2 displaying gene expression changes in heroin vs. saline groups, we also implemented EnrichR’s gene set databases PanglaoDB Augmented 2021, which is a single-cell RNA-sequencing database for identifying cell-type enrichment, and GO Biological Process 2021. Enrichments were ranked by adj.*p* value [26]. Multivariant plots display top ranked gene sets, Y-axis = rank from 1–5 for Cell-type and Gene ontology (GO BP) gene sets, plotted according to signed -log10(adj.*p* value) on the X-axis to indicate directionality of expression of genes analyzed, and the number of DEx genes overlapping with the gene set (bubble size), and the comparison source of DEx genes (fill color).

### Statistical analyses

For western blot comparisons, Student’s *t* tests were performed to compare saline vs. heroin conditions at different time points. For all behavioral testing involving more than two treatments and time points, Two-way ANOVAs were performed with subsequent Dunnett’s (for heroin SA escalation) or Tukey’s post hoc analyses for multiple comparisons (MC) (as indicated throughout the text). In molecular analyses, all animals used were included as separate *n*s (samples were not pooled). Significance was determined at *p* < 0.05. All data are represented as mean ± SEM.

## Results

### H3Q5dop is increased during both acute and prolonged abstinence in VTA following heroin SA

**Fig. 1:**
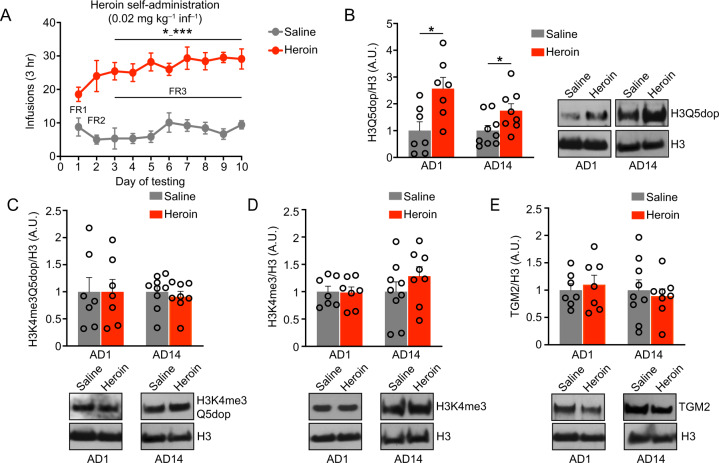
Histone H3 dopaminylation in VTA is dysregulated by heroin SA. **A** Number of infusions earned in daily 3 h (FR1 to FR3) test sessions in rats self-administering 0.02 mg/kg/infusion heroin or saline (*n* = 8/group) for 10 days (two-way RM ANOVA (F [2.348, 39.91] = 26.73, *p* < 0.0001), with Dunnett’s post hoc analysis vs. day 1, *p* = 0.03–0.0008). Western blot analysis of (**B**) H3Q5dop is significantly upregulated at AD1 via Student’s *t* test (*t* = 2.952, df = 12, *p* = 0.012), and AD14, *vi*a Student’s *t* test (*t* = 2.298, df = 15, *p* = 0.03) from heroin vs. saline SA rats (*n* = 7–9/group). **C** H3K4me3Q5dop, (**D**) H3K4me3 and (**E**) TGM2 in VTA tissues [abstinence day (AD) 1 or 14] from heroin vs. saline SA rats show no significant difference (*p* > 0.05). All blots were normalized to total H3 as a loading control. Data presented as *p ≤ 0.05, ***p ≤ 0.001, average ± SEM.

Given that histone dopaminylation (H3Q5dop), but not combinatorial H3 lysine 4 (K4) trimethyl (me3) Q5dop, was previously found to accumulate in VTA during prolonged, but not acute, abstinence from cocaine SA [11], we hypothesized that chronic exposures to heroin may similarly alter the deposition of H3Q5dop in this brain region. To assess this, we employed a heroin SA regimen wherein rats received i.v. infusions of heroin or saline (as a non-reinforcement control) paired with a light cue (Fig. 1A) for a period of 10 d, during which they escalated their heroin intake (Two-Way ANOVA, effect of time x condition F [9, 144] = 2.573, *P* = 0.0089, followed by Dunnett’s MC test for d1 vs. d5-d10; *p* = 0.03-*p* = 0.0008) [15]. Following SA, rats underwent forced abstinence in their home cages for 1 d vs. 14 d, the latter of which represents a time period where cue-induced heroin seeking is expressed [27]. On abstinence day (AD) 1 or AD14, VTA tissues were collected and western blotting was performed to quantify histone dopaminylation levels (both H3Q5dop and H3K4me3Q5dop), as well as expression of an associated histone PTM (e.g., H3K4me3) and the writer Transglutaminase 2 enzyme. We found that H3Q5dop levels significantly increased (Fig. 1B) in VTA during acute abstinence (AD1) from heroin SA vs. saline SA, *via* Student’s *t* test (t = 2.952, df = 12, *p* = 0.012). This increase persisted to extended abstinence periods (AD14), via Student’s *t* test (*t* = 2.298, df = 15, *p* = 0.03). Neither H3K4me3Q5dop nor total H3K4me3 (Fig. 1C, D) showed increases at either time point (Student’s *t* test, *p* > 0.05). The writer enzyme for H3Q5dop was unaffected in its expression during periods of abstinence from heroin (Student’s *t* test, *p* > 0.05) (Fig. 1E). Together, these data indicate that accumulation of histone dopaminylation in VTA occurs during abstinence from heroin. Interestingly, heroin-induced accumulation of H3Q5dop was found to occur more rapidly after opioid exposure vs. cocaine, with significant increases detected as soon as AD1. This increase persisted to AD14, suggesting that H3Q5dop may contribute, at least in part, to the opioid-induced transcriptional and behavioral alterations underlying relapse-like behaviors associated with drug exposure.

### H3Q5dop regulates heroin associated gene expression programs during prolonged abstinence

**Fig. 2:**
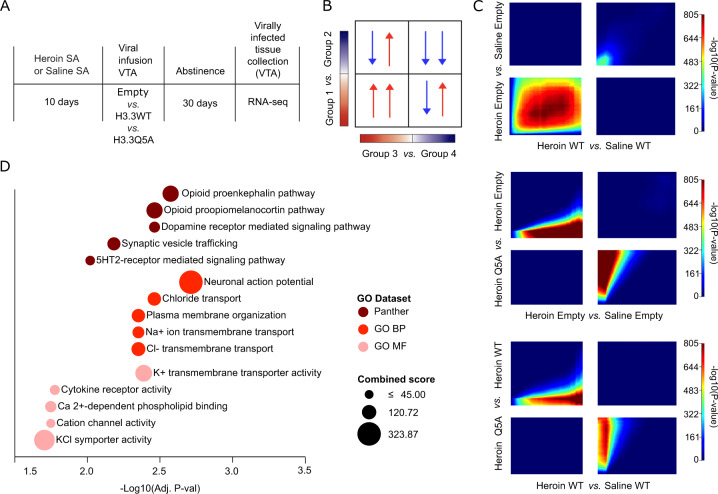
H3Q5dop in VTA contributes to heroin-mediated gene expression. **A** Experimental timeline of heroin SA (3 h @ 0.02 mgs/kg/infusion, FR1–3) vs. saline SA RNA-seq experiment following viral transduction with either empty vector or H3.3 WT vs. H3.3Q5A viruses (*n* = 7–8/group). **B** Schematic depicting Rank-rank hypergeometric (RRHO) plots, which allow for the comparison of two gene expression signatures, comprised of a ranked list based upon differential expression differences between two groups. Higher *p* values (indicated by color) in each of the four quadrants reflect the relationship of gene expression between comparisons. Bottom left indicates overlap between genes upregulated in group 1 vs. group 2 and genes upregulated in group 3 vs. group 4, and vice versa for top right. Top left indicates overlap between genes upregulated in group 1 vs. group 2 and genes downregulated in group 3 vs. group 4, and vice versa for bottom right. **C** RRHO plots depicting transcriptome-wide gene expression patterns indicate that Heroin Empty and Heroin H3.3 WT conditions show high degree of convergent expression (significant *p* values for overlap in bottom left quadrant, representing genes upregulated in both comparisons vs. respective saline groups), particularly for upregulated genes, while Heroin Q5A induces reversed gene expression patterns from both Heroin Empty and Heroin WT conditions (significant *p* values for overlap in top left and bottom left quadrants). Color heatmap displays -log10(P value) for significance of overlap. **D** GO Enrichment Analyses for the set of overlapping DEx genes shared between Heroin H3.3Q5A *vs*. Heroin Empty and vs. Heroin H3.3 WT. Multivariant bubble plots display the top enriched terms identified, Y-axis = rank for Panther, GO Biological Process, and GO Molecular Function gene sets, plotted according to -log10(adj.*p* value) (X-axis), and the combined score of the overlap (bubble size).

To explore the functional consequences of H3Q5dop accumulation during heroin abstinence, we used an AAV2 vector that expresses the H3 variant, H3.3, with a glutamine-to-alanine substitution at position 5 (H3.3Q5A), which cannot be dopaminylated. This H3.3Q5A construct has been shown to strongly express the mutant H3.3Q5A protein in a nuclear-specific manner in adult neurons, resulting in downregulation of H3Q5dop [11]. Importantly, we have previously shown that viral expression of this H3.3Q5A mutant prevents the accumulation of H3Q5dop observed after long-term abstinence from cocaine SA [11]. Therefore, in order to directly evaluate the impact of reducing H3Q5dop accumulation on gene expression programs during prolonged abstinence from heroin, we delivered one of three AAV viral vectors: H3.3QA; H3.3 wildtype (WT); or an empty GFP control vector [11, 12] intra-VTA, following heroin SA and a 30 d period of enforced abstinence (Fig. 2A), [11]. On AD30, infected VTA tissues were microdissected and processed for RNA-seq.

We first utilized a threshold free Rank-Rank Hypergeometric Overlap (RRHO) analysis [24] to characterize the relationship between gene expression patterns across the entire transcriptome for viral group comparisons (Fig. 2B). RRHO revealed a significant overlap in genes that are upregulated in both heroin empty vs. saline empty and in heroin H3.3 WT vs. saline H3.3 WT (Fig. 2C top panel), indicating that these two heroin SA conditions showed highly similar increases in gene expression compared to corresponding saline groups. In order to determine effects of the H3.3Q5A mutant in the context of heroin, we plotted the transcriptome of heroin H3.3Q5a *vs*. both heroin empty and heroin H3.3 WT vectors (Fig. 2C*,* middle and bottom panels). For both comparisons, we identified a robust pattern of anti-correlation, demonstrating that the H3.3Q5A mutant induced reversed gene expression patterns compared to heroin empty vector and heroin H3.3 WT groups.

To profile specific heroin-induced gene expression changes, we employed pairwise comparisons to identify differentially expressed (DEx) genes between heroin groups (empty vector and H3.3 WT) vs. saline groups (empty vector and H3.3 WT), defined at a false discovery rate (FDR) of <0.05, log2FC ± 0.2. We identified 736 upregulated and 1005 downregulated DEx genes between the heroin empty vs. saline empty conditions, and 303 upregulated and 823 downregulated DEx genes between heroin H3.3 WT and saline H3.3 WT comparisons, respectively (SI Appendix, Table S1). There were only 29 DEx genes between empty vs. H3.3 WT, confirming that the majority of gene expression changes induced by the H3.3Q5A mutant are not due to exogenous expression of the histone H3.3 protein. Importantly, reducing levels of H3Q5dop with the mutant H3.3Q5A virus reversed over 50% of gene expression changes induced by extended heroin SA abstinence, compared to both heroin empty and heroin H3.3 WT groups (SI Appendix, Fig. S1*A-B*).

In order to determine which pathways were affected by blocking H3Q5dop, we next performed a Gene Ontology Enrichment Analysis (GO) on the set of overlapping DEx genes (*n* = 321) that were reversed between heroin H3.3Q5A vs. heroin empty and heroin H3.3 WT conditions (SI Appendix, Fig. S1C). We found significant associations with genes involved in opioid neuropeptide pathways, dopamine receptor signaling, cytokine responses, synaptic transmission, and regulation of neuronal action potentials (Fig. 2C), indicating that H3Q5dop accumulation during heroin abstinence may drive specific neuroadaptations related to altered plasticity in the VTA.

Interestingly, while levels of the H3Q5dop PTM itself appear to renormalize by AD30 (SI Appendix, Fig. S2), our RNA-seq data reveals broad transcriptional changes (~800 DEx genes) at this time point as a result of blocking dopaminylation with the AAV H3.3Q5A virus throughout heroin abstinence, with expression starting at approximately AD7 and peaking at AD28 [28]. This indicates that H3Q5dop accumulation during the course of abstinence drives substantial and persistent molecular changes in this region that precipitate long-lasting alterations in VTA function. Although the VTA is a critical source of dopaminergic neurotransmission to the ventral striatum, this region had surprisingly not yet been profiled at the transcriptomic level in a volitional animal model of heroin dependence. Therefore, we also performed GO analyses of DEx genes between heroin vs. saline groups, which revealed that extended abstinence from heroin induced long-term changes in VTA interneurons, as well as in the generation of neuronal action potentials, myelination machinery, oligodendrocyte maturation, and ECM-based remodeling and plasticity (SI Appendix, Fig. S3).

To determine if extended heroin vs. cocaine abstinence induced similar gene expression changes in the VTA, we performed odds ratio analyses to measure the extent of overlap of DEx genes in both conditions, using our previously published cocaine dataset [11]. Interestingly, we identified a significant pattern of opposing gene expression, with robust overlaps identified between genes that were upregulated in heroin and downregulated in cocaine (and vice versa) (SI Appendix, Fig. S4A), suggesting that these two substances likely act on similar pathways but in opposite directions within the VTA. Furthermore, we also found that gene expression reversals induced by H3.3Q5A also displayed opposite directionality between heroin vs. cocaine (SI Appendix, Fig. S4B), indicating that H3Q5dop likely functions as a mediator of general neural plasticity within this brain region, rather than as a downstream effector of a specific drug-induced pathway.

### Accumulation of H3Q5dop in VTA contributes to heroin-seeking after extended abstinence

**Fig. 3:**
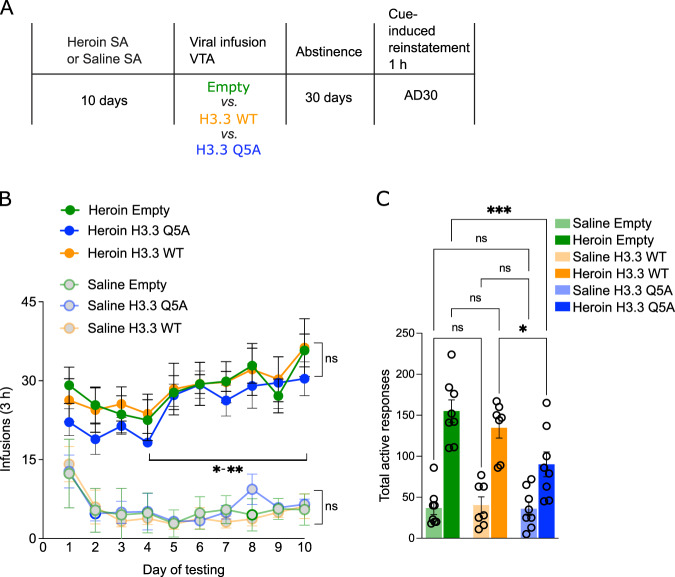
Reducing H3Q5dop in VTA attenuates heroin-seeking. **A** Experimental timeline of heroin SA (3 h @ 0.02 mgs/kg/infusion, FR1–3) vs. saline SA drug-induced reinstatement experiments following viral transduction with either empty vector, H3.3 WT or H3.3Q5A viruses. **B** Rats were put through 10 days of heroin SA, with escalation of intake, *n* = 7–8/group, via two-way ANOVA, with significant effect of time (F [9, 180] = 6.512, *p* < 0.0001), and no effect of virus (*p* = 0.53), followed by Dunnett’s MC test for day 4 vs. day 10 (*p* = 0.01-*p* = 0.003). There was no significant difference in heroin intake between animals assigned to different viral groups. There was no significant escalation of intake for saline groups (*p* = 0.9). Rats were infected intra-VTA with one of the three viral vectors (on day 11), followed by 30 d of withdrawal, extinction and (**C**) Cue-induced reinstatement of drug seeking (*n* = 7–8/group); the total number of active responses was reduced in H3.3Q5A rats vs. H3.3 WT (*p* = 0.028) and empty vector controls via two-way ANOVA, with a significant effects of heroin (F [1,40] = 86.82, *p* = <0.0001), virus (F [2,40] = 4.456, *p* = 0.017), and interaction (F [2,40] = 4.01, *p* = 0.025). Tukey’s MC tests revealed significant differences between heroin H3.3Q5A vs. heroin empty (*p* = 0.0007) and heroin H3.3Q5A vs. heroin H3.3 WT (*p* = 0.0288), and no significant effects between saline viral groups (*p* > 0.05). For all graphs, ^#^*p* < 0.1, *p < 0.05, **p < 0.01, ***p < 0.001. Data presented as average ± SEM.

Finally, to investigate the consequences of reducing H3Q5dop levels on heroin relapse-relevant behaviors, we put an independent cohort of animals through chronic heroin self-administration, followed by intra-VTA viral infusions on AD1, using the same three viral vectors discussed above (Fig. 3A, B). On AD30, animals were tested for heroin-seeking behavior in a cue-induced reinstatement paradigm. Preventing H3Q5dop accumulation in heroin SA animals significantly reduced motivated heroin-seeking behaviors in heroin animals (Fig. 3C) via Two-way ANOVA, with significant effects of heroin (F [1,40] = 86.82, *p* = <0.0001), virus (F [2,40] = 4.456, *p* = 0.017), and interaction (F [2,40] = 4.01, *p* = 0.025) observed. Tukey’s MC tests revealed significant differences between heroin H3.3 Q5A vs. heroin empty (*p* = 0.0007) and heroin H3.3Q5A vs. heroin H3.3 WT (*p* = 0.0288), and no significant effects between saline viral groups (*p* > 0.05). Importantly, we have previously shown that this H3.3Q5A mutant histone does not affect operant behavior for natural rewards [11]. Together, these data indicate that aberrant accumulation of H3 dopaminylation in VTA promotes a discrete transcriptional program capable of mediating relapse associated vulnerability across pharmacologically distinct addictive substances.

## Discussion

As part of its mechanism of action, heroin exposures increase neurotransmission from dopaminergic neurons in VTA to reward-processing projection regions in striatum, among other neuronal regions. These fluctuations in dopamine signaling are thought to contribute to maladaptive plasticity and impaired decision-making observed during substance dependence. However, the mechanisms that mediate these processes at the epigenetic level are not fully characterized. Here, we showed that H3Q5dop rapidly accumulates in rodent VTA at 24 h post-heroin SA, and that this increase persisted during protracted periods of heroin abstinence (e.g., AD14). We next profiled gene expression changes that occurred after attenuating levels of H3Q5dop during heroin abstinence and identified highly significant overlap with regulation of opioid and dopaminergic signaling, cytokine pathways, and synaptic transmission/regulation of neuronal action potential related processes. Finally, these transcriptomic changes coincided with a significant decrease in drug-seeking behavior after 30 d of heroin abstinence. Together, these data indicate that H3Q5dop plays a critical role in promoting persistent functional alterations that drive maladaptive behaviors related to heroin relapse vulnerability.

Our findings also highlight the critical role of H3Q5dop in regulating epigenetic changes after chronic exposures to, and abstinence from, different drugs of abuse (e.g., an opioid vs. a psychostimulant). However, we also observed key differences between the substances with respect to both the dynamics and gene targets of H3Q5dop. First, we found that H3Q5dop is significantly increased in rodent VTA as soon as AD1 post-heroin SA, an increase that persists throughout more extended periods of abstinence. In contrast, levels of H3Q5dop during cocaine abstinence remained unchanged 24 h after the last day of cocaine SA, and then slowly accumulated during long-term withdrawal (e.g., AD30; a time point in which H3Q5dop is no longer globally elevated following heroin exposures). This temporal difference in H3Q5dop dynamics may reflect differences in dopaminergic activity in response to either substance, particularly during more acute periods of abstinence—for example, electrophysiology and fiber photometry studies have demonstrated that while cocaine exposures lead to immediate inhibition of dopaminergic VTA neurons, heroin exposures strongly activate these projections [29]. Furthermore, neurons in striatum activate distinct pathways in downstream projection regions in response to heroin vs. cocaine, suggesting that the reward system may display circuit-specific responses to different classes of drugs of abuse [30, 31].

Another major finding revealed in our current study relates to VTA gene expression changes mediated by H3Q5dop in response to heroin vs. cocaine SA. Our RNA-seq analyses found that heroin abstinence induced robust changes in genes involved with interneuron transmission activity, oligodendrocyte myelination, and ECM remodeling. These transcriptomic changes correlate to current hypotheses regarding opiate disinhibition of GABAergic regulation of dopaminergic activity through local interneuron networks in the VTA, thereby leading to increased DA output from the VTA [32, 33]. These findings also parallel our previous work demonstrating that exposures to heroin increase expression of oligodendrocyte genes and myelination pathways in the PFC, a phenomena that has been confirmed in recent single-cell RNA-seq studies [34, 35]. Interestingly, we also found that heroin and cocaine appear to elicit significantly opposite patterns of gene expression in the VTA, which has also been observed in other brain regions within the mesolimbic reward pathway in human tissues, and in preclinical models [36, 37]. Our findings suggest that while both cocaine and heroin dependence converge on similar critical molecular pathways in the VTA, these substances alter reward circuitry function by driving gene expression in opposing directions.

Importantly, reducing accumulation of H3Q5dop with a dominant negative H3.3 mutant virus led to specific gene expression changes in molecular processes mediating action potential generation, membrane conductance, and synaptic regulation, as well as signaling cascades directly related to opioid and dopaminergic pathways. The renormalization of these gene expression alterations led to significantly reduced drug-seeking behavior following extended abstinence from heroin SA, suggesting that these pathways contribute to relapse vulnerability in drug dependence. Together, our data show that H3Qdop accumulation may regulate a state of increased neuronal plasticity, derived from aberrant signals that disrupt the balance of excitatory/inhibitory modulation of VTA activity. Given that chronic heroin use followed by withdrawal induces adaptations in dopaminergic neurotransmission and changes in membrane ion conductance and ion channels [38], it is notable that reducing H3Q5dop acts to specifically alter these pathways, indicating that this modification may actively participate in the molecular translation of heroin withdrawal to altered neural activity.

Finally, our previous work established that the H3Q5dop modification may be an upstream epigenetic regulator of dopaminergic neurotransmission in drug dependence. Our current findings suggest that manipulation of H3Q5dop during different stages of drug dependence may represent a useful tool to reveal important gene expression profiles that are unique to specific classes of substances. Moving forward, delineating specific transcriptional signatures that effectively promote reward-circuit remodeling for different drugs of abuse may provide insights to guide clinicians in tailoring therapeutic strategies towards the unique mechanisms of action for a given drug class. For example, substances like nicotine and alcohol, which also converge on promoting dopamine outflow, have distinct molecular interactions with cellular substrates in the VTA. Further study of H3Q5dop dynamics in the context of these different substances may reveal distinct epigenetic mechanisms that translate their respective receptor-binding signals into increased plasticity. Importantly, recent reports of sex-specific dopamine activity after heroin exposure and increased heroin-seeking behaviors in females highlight the need to expand investigations of H3Q5dop mechanisms in female subjects [39]. Overall, the data presented here demonstrate that heroin acts through H3Q5dop deposition as an epigenetic mechanism in the VTA to recruit specific transcriptional programs that alter dopaminergic neurotransmission and drive drug-seeking behaviors during prolonged abstinence.

## Supplementary information

Supplemental Material

Extended Data Table 1

## Data Availability

Data from RNA-seq experiments have been deposited in the National Center for Biotechnology Information Gene Expression Omnibus (GEO) database under accession number GSE169628. We declare that the data supporting findings for this study are available within the article. No restrictions on data availability apply.
