# Supplementary material for: Histone H3 dopaminylation in ventral tegmental area underlies heroin-induced transcriptional and behavioral plasticity in male rats
Source: Neuropsychopharmacology. 2022 Jan 29;47(10):1776–83. doi: 10.1038/s41386-022-01279-4 (PMC9372029; doi:10.1038/s41386-022-01279-4)
Supplement: Supplementary file 1 — Supplemental Material [file 41386_2022_1279_MOESM1_ESM.pdf]

SUPPLEMENTAL MATERIAL

Histone H3 dopaminylation in ventral tegmental area underlies heroin-induced transcriptional and behavioral plasticity in male rats

Running Title: *H3Q5dop in VTA mediates heroin-seeking*

Sasha L. Fulton[1,5], Swarup Mitra[2,5], Ashley E. Lepack[1], Jennifer A. Martin[2], Andrew F. Stewart[1], Jacob Converse[2], Mason Hochstetler[2], David M. Dietz[2*], Ian Maze[1,3-4*]

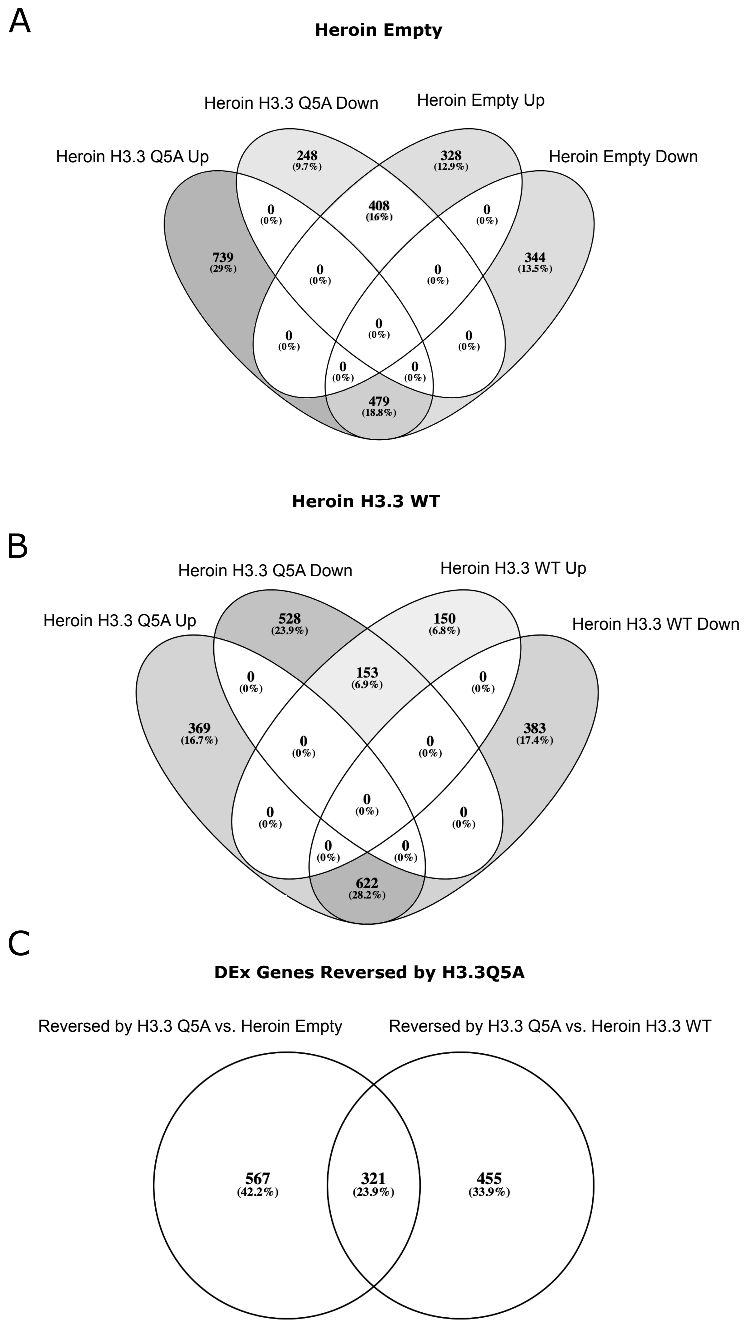

Figure S1. Overlapping DEx genes reveals shared upregulated and downregulated genes in heroin *vs.* saline

Venn diagram showing DEx genes upregulated and down regulated between heroin H3.3Q5A groups and (**A**) heroin empty *vs.* saline empty groups and (**B**) heroin H3.3WT *vs.* saline H3.3 WT groups reveals that H3.3Q5A reverses over 50% of heroin-induced

gene expression changes.. (C) Venny diagram showing overlap of all DEx genes between heroin H3.3Q5A *vs*. heroin empty or *vs.* heroin H3.3WT.

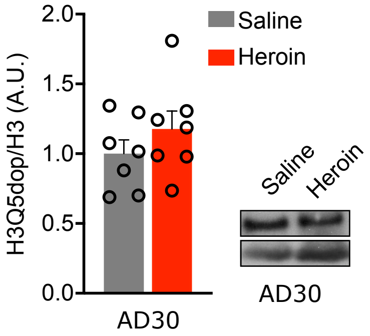

Figure S2. Levels of H3Q5dop in the rat VTA renormalize by AD30

H3Q5dop is not differentially regulated at AD30, *via* Student's t-test ($t=1.099$, $df=12$, $p=.29$).

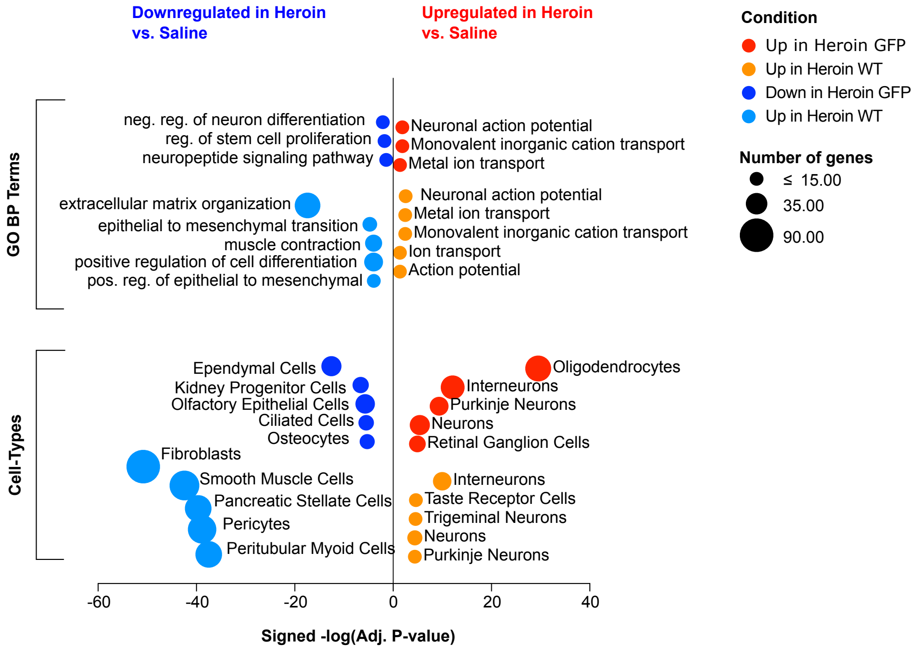

Figure S3. GO analysis reveals biological pathways and cell-types associated with DEx genes in heroin *vs.* saline

Bubble plot displays GO analysis for DEx genes between heroin *vs*. saline groups. Enrichments are ranked by adj. p-value [25]. Multivariant plots display top ranked gene sets, Y-axis = rank from 1-5 for Cell-type (PanglaoDB Augmented 2021) and Gene ontology (GO BP) gene sets, plotted according to signed -log10(adj.p-value) on the X-axis to indicate directionality of expression of genes analyzed, and the number of DEx genes overlapping with the gene set (bubble size), and the comparison source of DEx genes (fill color).

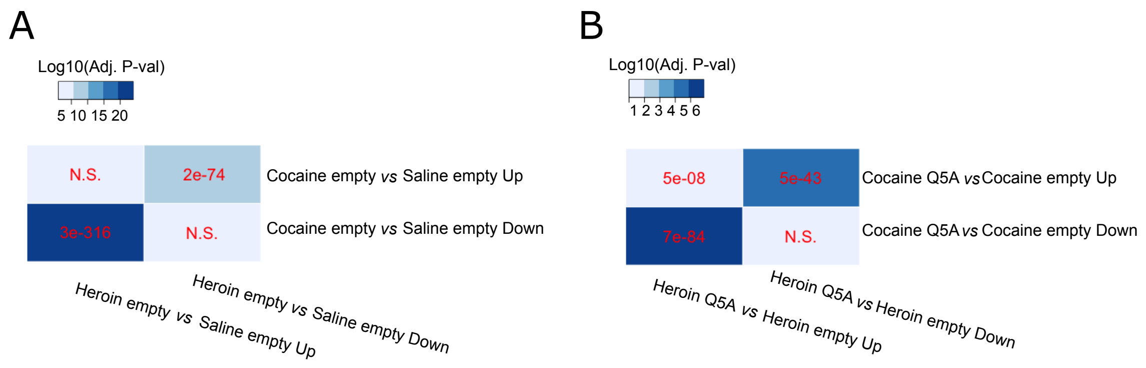

Figure S4. Heroin and cocaine regulate similar gene expression pathways in opposite directions.

Odds ratio analysis reveals heroin and cocaine regulate gene expression in opposite directions. Plots display adj. p-val for the overlap between DEx genes in (**A**) heroin empty vs. saline empty and cocaine empty vs. saline empty and (**B**) heroin H3.3Q5A vs. heroin empty and cocaine H3.3Q5A vs. cocaine empty.

Supplementary Data **Table S1** (Processed RNA-seq Data; Excel Format)
